# Diagnostic imaging versus surgical procedure: intra- and postoperative OCT evaluation of sutureless scleral-fixated intraocular lens implantation and possible related complications

**DOI:** 10.1007/s00417-021-05087-2

**Published:** 2021-03-12

**Authors:** Raffaele Nuzzi, Alessandro Rossi

**Affiliations:** Institute of Ophthalmology, Department of Surgical Sciences, University of Turin, Via Cherasco, 23 10126 Turin, Italy

**Keywords:** Anterior Segment OCT (AS-OCT), Anterior Segment diagnostic imaging, Sutureless scleral-fixated intraocular lens, Cataract surgery, Cataract complications

## Abstract

Because the popularity of corneal refractive surgery has been increasing throughout the last 25 years, many authors have thought to apply optical coherence tomography (OCT) to the anterior segment (AS-OCT); by revising the instrumentation needed and slightly improve the technique, it has become an element of vital importance in order to ensure a complete and exhaustive pre- and postsurgical evaluation. Many applications of OCT have been recently developed—mostly in cataract surgery due to the increasing numbers—such as chamber biometry, which is used in a preoperative stage to determine the details of IOL implantation, and lens evaluation. The aim of this review is to assess the applications of anterior segment OCT in dislocated IOL and/or capsular bag exchange surgery with scleral sutureless fixated intraocular lens and monitoring of possible postoperative complications.

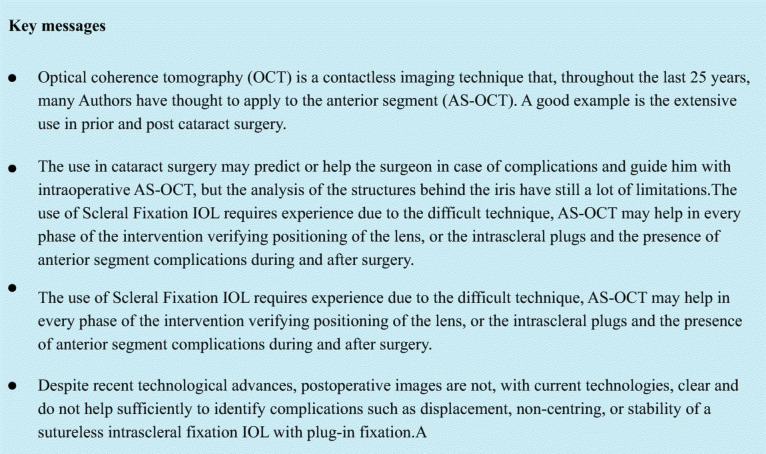

## Introduction

Optical coherence tomography (OCT) is a contactless imaging technique that provides cross-sectional images of the internal structures of the eye [1]. Because the popularity of corneal refractive surgery has been increasing throughout the last 25 years, many authors have thought to apply such a detailed technique to the anterior segment; by revising the instrumentation needed and slightly improve the technique, it is possible to perform an anterior segment OCT (AS-OCT). The application on corneal pathology and surgery has determined the greatest thrust in technological advancement, guaranteeing an ever more detailed investigation both before and after PKP, DALK, DSAEK, DMEK and LASIK. The addition of the data from the endothelial cell count by biomicroscopy has made it possible to combine quantitative data, for example, in cases of corneal decompensation, following the various operations that can be performed in patients with cataract. Furthermore, many other applications of OCT have been recently developed, such as angle evaluation (in order to diagnose narrow-angle glaucoma) and anterior chamber biometry (which is used in a preoperative stage to determine the details of IOL implantation) [2]. The aim of this review is to assess the applications of anterior segment OCT in dislocated IOL and/or capsular bag exchange surgery with scleral sutureless fixated intraocular lens and monitoring of possible postoperative complications.

## Main text

## AS-OCT: how does it work

Optical coherence tomography (OCT) is a contactless technique which analyses the ocular structures through low coherence interferometry [1]. Since its development, OCT imaging has become an essential element of the cornea and anterior eye segment evaluation. Because OCT’s technology has been improving (for example, the speed and resolution of the images captured have improved), its impact on clinical practice is consistent. In addition to this, the processing software allows the analysis of multiple scans, the development of 3-dimensional reconstruction and the acquisition of extremely accurate measurements. Thus, this newly improved technology is of vital importance during all the operative phases: in the preoperative phase, it allows an accurate planning of the surgery; in the intraoperative phase, the acquisition of real-time imaging may affect surgical decision; and during the postoperative phase, it allows the evaluation of surgical outcomes and possible complications.

Optical coherence tomography measures the echo time delay of the light backscattered from tissue structures, thus generating 2- or 3-dimensional tomographic images. A beam splitter splits the light coming from a low-coherence light source in two parts, thus directing them into the two arms of an interferometer. In the reference arm, the light is reflected by a mirror; in the sample arm, the light is back scattered by the tissues. The illumination properties (i.e. shape, depth of focus and intensity distribution of the beam) are defined by the optical components in the sample arms; different tissues have different refractive index. The light returning from both arms are recombined at the beam splitter and redirected towards the detector.

When the pathway between the two beams is within the coherence length of the light source, interferences occur. To maximize depth resolution, Time-domain (TD) or Fourier-domain (FD) OCT have been developed. TD OCT is characterized by a reference delay whilst translating the mirror in the reference arm. A point detector collects the light, and depth resolution is examined whilst the mirror moves at a constant speed. A complete travel of the reference mirror is called A-scan. Whilst TD OCT detects the light echoes sequentially, FD OCT collects technique modulations of the source spectrum, capturing simultaneously all the spectral components. After that, the light is spectrally analysed through the use of a spectrometer and a CCD line camera; because the reference arm’s mirror is static, an A-scan is obtained with one camera shot. This leads to higher acquisition rates. Thus, the read-out rate of the line sensor limits the acquisition speed of spectrometer-based systems. It is then an inverse Fourier transform that transmutes the wave number-dependent signals into axial scan information. In addition to this, some FD OCT technologies are based on swept-source (SS) technology: the wavelength is rapidly swept through light source with the aid of a point detector. This leads to acquisition rates that might reach up to several MHz.

## AS-OCT: current uses in cataract surgery

Because cataract surgery has recently reached successful outcomes, the patients have great expectations on the operation, thus placing the surgeon under a heavy burden [3]. Recently, in literature, many authors have underlined the usefulness of anterior segment OCT (AS-OCT) in cataract surgery: it allows the surgeon to perform an accurate preoperative planning by evaluating the conditions of the surfaces [4], by calculating the IOL power [5], by evaluating the anterior chamber structures [6, 7] and by assessing the presence of possible risk factors that may lead to intraoperative or postoperative complications [8]. Furthermore, intraoperative use of AS-OCT has been used to evaluate in vivo clear cornea wound architecture [9, 10] and OCT-guided femtosecond laser-assisted cataract surgery [11]. In addition to this, OCT plays a fundamental role in the postoperative setting such as the assessment and the management of postoperative complications [12] the assessment of stability or optical changes [13, 17], the evaluation of a postoperative laser-assisted in situ keratomileusis (LASIK) flap [15].

AS-OCT might also be carried out intraoperatively. Modern technology and modern surgical instrumentation comprehend different guiding systems that support the surgeon’s precision. Within such modern features, operating microscopes ensure real-time, high-resolution evaluation of corneal thickness along with the lens’ position, iris boundaries, iridocorneal angles and ciliary body. The recent technological boost has been largely supported by the development of new laser-assisted cataract surgery techniques. During the latter, the mathematical calculations processed by Fourier-domain OCT provide a three-dimensional high-resolution reconstruction of the ocular structure and accurate image guidance, thus improving the precision and safety of laser cataract surgery [16]. The most used pieces of machinery used for these purposes are LenSx, Catalys and VICTUS systems. Palanker et al. [11] report promising data through OCT-guided femtosecond laser-assisted cataract surgery, to confirm the real need of technologic support. Further studies are to be made to provide the cost-effectiveness and risk–benefit evaluation of femtosecond laser-assisted cataract surgery compared with traditional phacoemulsification.

It is of vital importance to underline that a suboptimal size selection of the lens may result in IOL dislocation, pupil ovalization, chronic inflammation or endothelial cell loss [17]. The great variety of patients that undergo cataract surgery might not entirely reflect the populations that have been accurately selected to carry out studies on the effectiveness of the new surgical techniques; the comorbidities, the age and the anatomical variants are to be considered. The studies that analyse the support that modern technology might offer to the surgeon must concentrate on different clusters of patients, because those that are affected by other comorbidities or that might develop complications are those that might benefit the most. The IOL’s instability, in fact, is a short-medium term complication that has been increasing in the last few years, because of the improved survival of the patients that undergo surgery and of the use of ultrasounds which is increasingly aggressive. In addition to this, myopic patients must be taken in serious considerations due to the fact that they frequently tend to develop surgical complications.

The most recent examples of in vivo postoperative evaluation of the position of the lens and its dioptric power have used AS-OCT to precisely assess the accuracy of the surgery performed. Jeorge L Aliò et al. [13] evaluated the stability of angle-supported IOL with photopic miosis and pharmacologic mydriasis using AS-OCT. The authors claim that AS-OCT is able to provide a reliable measurement of ACD, and iridocorneal angles. Kumar et al. [18] used AS-OCT to evaluate IOL tilt in relation to the limbus, reporting a non-significant tilt. Pe´rez-Cambrodı´ et al. [19] evaluated in vivo AS-OCT data which suggested that the phakic refractive lens (PRL; Carl Zeiss Meditec) have a tendency to nasal position, thus altering the visual outcome of the surgery, especially in deep anterior chambers. AS-OCT has been also used to assess capsular closure after cataract surgery [14, 20], to evaluate in vivo the IOL’s effects on the posterior capsular which might cause subsequent opacification. Werner et al. [20] reported that AS-OCT is of great usefulness in assessing the presence, location and density of postoperative ocular media opacities. The authors concluded that, by determining the causes of media opacity through AS-OCT, many unnecessary procedures might be avoided.

Cataract surgery and AS-OCT are inevitably linked; the use of diagnostic technology is of great usefulness to the surgeon. Pho Nguyen and Vikas Chopra [21] have thoroughly analysed the current uses and future research prospectives that might be taken into consideration. Nevertheless, there are many limits to the applications of modern technology, which are discussed in the following paragraph, that underline how the attention of scientific research should focus on the long-term complications of cataract surgery.

## AS-OCT: limits and analysis of the structures behind the iris

Despite the numerous technological advances, the greatest limit of preoperative, intraoperative and postoperative AS-OCT is that it does not allow the analysis of the structures that lie behind the iris [22]. The latter, in fact, cannot be clinically assessed because it is always hidden by the iris, no matter its dilatation; for the same reason, it cannot be assessed whilst surgery is being performed [22].

**Fig. 1:**
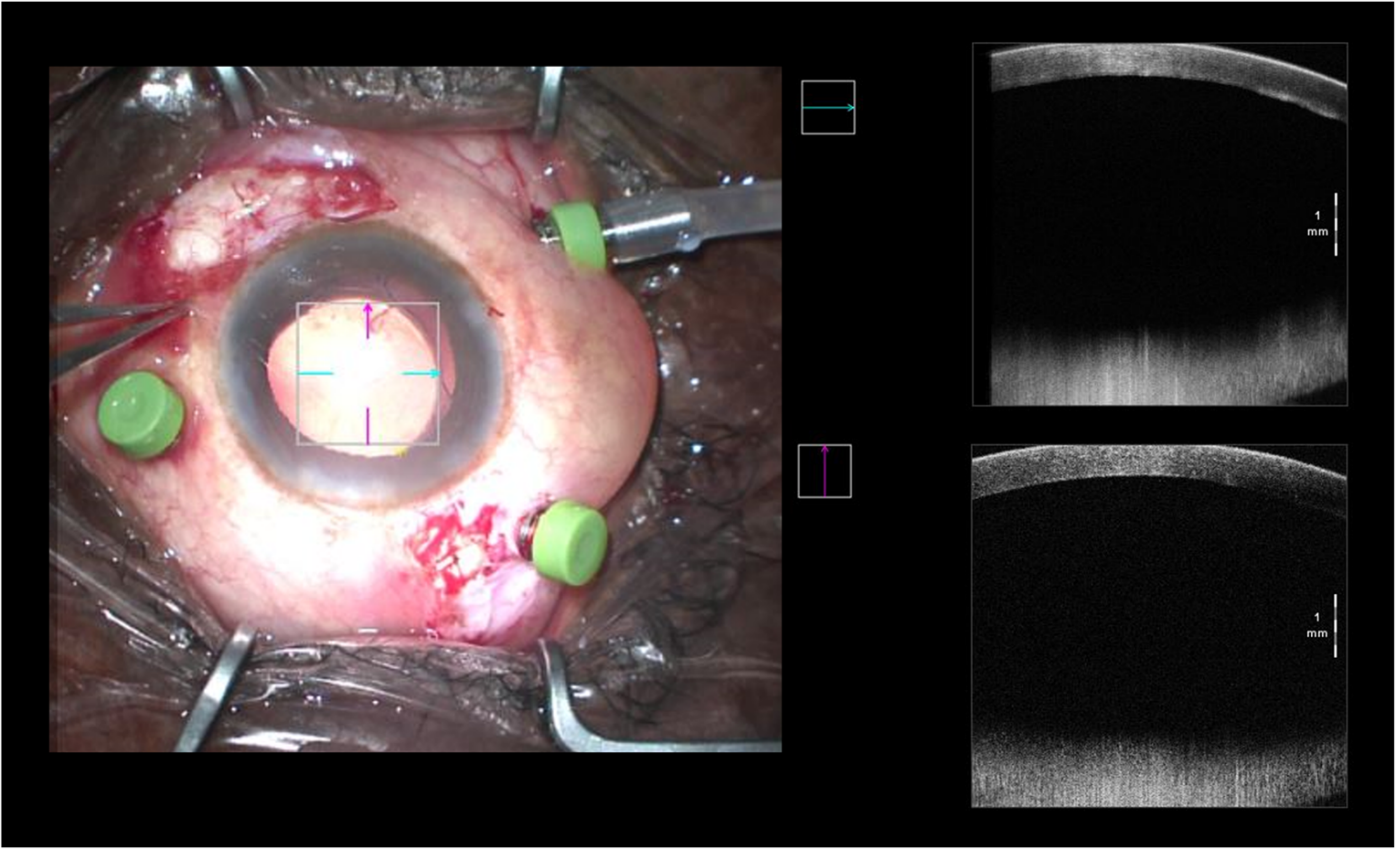
Intraoperative analysis of anterior segment, IOL position is not detectable

**Fig. 2:**
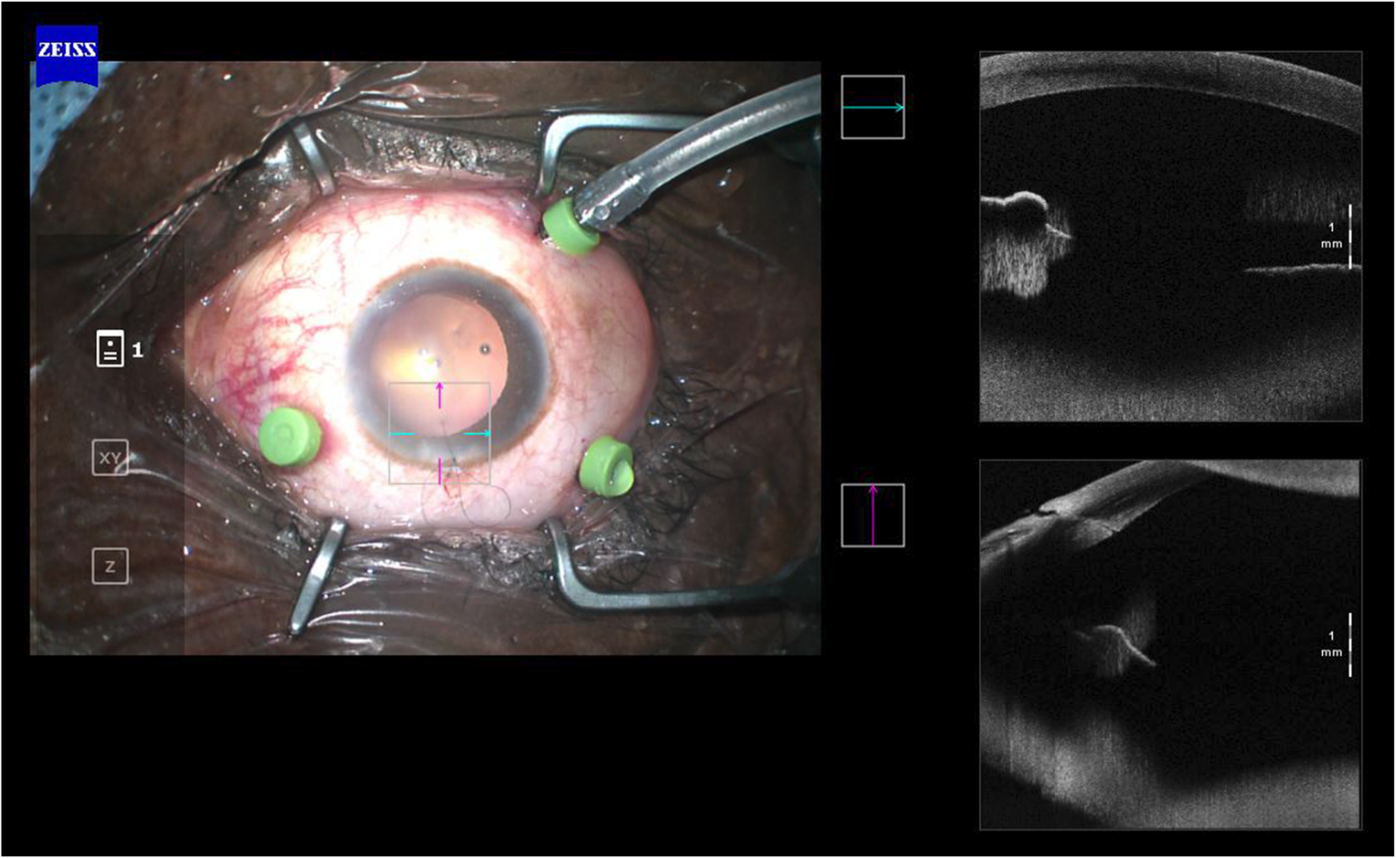
Surgically aphakic patient, no difference in intraoperative AS-OCT with a pseudophakic patient

Thus, given the reflectance properties of the lens that are currently in use, the position of the haptics and the insertion of the lens might only be supposed during each surgical phase; not even OCT is able to provide further information regarding this surgical site [23]. In fact, its rays are blocked by the iris. It is possible to clearly analyse only the central part, which, despite the grade of mydriasis achieved, will never cover the whole lens that has been inserted. In addition to this, when taking into consideration a posterior chamber lens, or one that has been inserted in the ciliary sulcus, it is not possible to analyse the insertion of the haptics. On the contrary, it is said that, in some cases, the position of the haptics might be analysed whilst the eye is in wide mydriasis with a single piece acrylic IOLs; of course, this possibility is directly correlated to the width of the corneal access [24] (Figs. 1 and 2).

Posterior chamber mono or multifocal lenses cannot be verified in a precise and unique way; in particular, should posterior capsule rupture occur, the lenses that are positioned in the ciliary sulcus cannot be assessed. Thus, AS-OCT loses its usefulness after surgery is performed; in fact, whilst the anterior chamber is always easily assessed, the posterior chamber is never accessible [25].

In addition to this, because the implanted lens is characterized by high transparency, it is even more difficult to detect through the use of this technique which analyses the acrylic thickness of the central part of the lens; in fact, it allows the assessment of the current position of the lens, but it does not allow the assessment of its correct position in the posterior chamber.

## Sutureless scleral-fixated intraocular lens implantation (SFIOL): surgical prerogatives

One of the serious complications of cataract surgery is late dislocation of the intraocular lens (IOL) [26]. Such condition can be managed through different techniques such as exchange or reposition of the dislocated lens, which may be carried out either with a pars plana vitrectomy or an anterior vitrectomy [27, 28]. In some cases, the lens can be sutured to the sclera or iris. The choice of the most appropriate technique depends on the surgeon. Normally, posterior segment surgeons choose pars plana vitrectomy followed by the repositioning of the lens [27, 29]; on the other hand, anterior segment surgeons go for an anterior vitrectomy followed by limbal incision and replacement of the IOL [28, 30]. Nevertheless, there are some difficulties that must be taken into consideration: nowadays, most of the IOLs consist of a single acrylic piece. Because of this, they are not suitable for being repositioned and sutured to the sclera or iris unless capsular support is adequately ensured. Therefore, a decrease in the repositioning procedures and a rapid increase of the replacement technique have been recently recorded [31].

The latter technique has been developed in order to avoid the need of ocular sutures which often lead to serious complications such as inflammation or infection and IOL dislocation or subluxation due to suture degradation or suture breakage. Sutureless intrascleral fixation techniques rely on intrascleral haptic fixation in order to ensure stability of the IOL [32]. Various techniques have been developed to obtain haptic fixation that can be obtained by creating a scleral flap or a scleral tunnel [33]. In order to carry out the great majority of these techniques, it is essential to dispose of special forceps that enable the surgeon to clutch the lens’ haptics, put them in place and then externalize them through sclerotomy.

Finally, an internalization of the haptics in intrascleral pockets previously made is performed; this avoids direct contact between the conjunctiva and possible inflammatory reaction or wound dehiscence.

## Sutureless scleral-fixated intraocular lens implantation (SFIOL): the role of anterior segment OCT in SFIOL surgery

Because there are different techniques, and at the same time different types on lenses, it is up to the surgeon to choose one of each, according to both his preferences and the patient’s characteristics. Thus, it is of vital importance that an intraoperative checking method is established in order to analyse the outcome of the surgery performed and to confront in an objective way the pre- and postoperative data. This may be obtained by making use of a preoperative, intraoperative and postoperative OCT, focusing on both the anterior and posterior segments. The imaging techniques that aim to analyse the anterior and posterior segment of the eye provide the surgeon with a three-dimensional view of the patient’s eye and make it easier to confront the condition of the structures before and after surgery is performed. Therefore, as an abdomen surgeon analyses CT images before surgery, so does the ophthalmologist that can evaluate and choose the best technique for the patient according to the images he analyses.

The logging of the pre-intra-postoperative data allows an easy consult and the possibility to share the images obtained. This is in line with the onset of technology in modern medicine. In fact, modern telemedicine protocols promote the freedom of the patient regarding the choice of the structure in which they prefer to undergo surgery or carry out their follow up examinations; some patients might even choose to lean for a virtual consult with the surgeon who has performed or will perform surgery, without the need of further travelling.

To the best of our knowledge, this is the first study in literature that evaluates the use of intraoperative OCT for the placing of a sutureless intrascleral fixation IOL with plug-in fixation. In addition to this, it is the first work that confronts the pre-intra- and postoperative images of the anterior eye segment; this implies the cooperation of the clinician and the surgeon in order to ensure the best management of the patient’s disease.

The use of a sutureless intrascleral fixation IOL is becoming more frequent [33], and the different surgical techniques are gaining in popularity. Such increase in interest on the matter is closely monitored by the lenses’ manufacturers that aim to propose new solutions [34] in order to ease and ensure the lens’ stability by improving the ergonomics and the haptics of three-dimensional structure. The development of new techniques and the manufacturing of more practical lens allow a wider spread of this surgical procedure [32].

According to some studies, the application of anterior segment-optical coherence tomography (AS-OCT) has proven to be of great use during the preoperative phase of cataract surgery, for instance, to analyse the anterior segment structures [35, 36]. The development of a 3D reconstruction system allows on one hand the objectification of the examination [37] and, on the other, the full knowledge of the pathological condition of the patient’s eye [38]. The surgeon, helped by a graphic representation of what he will be facing, can foresee possible intraoperative complications related to the single patient and eventually modify the technique procedures in order to avoid them. In fact, cataract surgery is very repetitive and thus subjected to unexpected complications [3]. On the contrary, the juxtaposition of a sutureless intrascleral fixation IOL requires more expertise and 360° mastery of eye surgery.

When a SFIOL is implanted, it is necessary to evaluate both the anterior and the posterior segments. The former is especially involved during the extraction of the dislocated lens or that of the cataract; the latter is involved when the lens haptics are exposed through scleral site. Thus, the surgical site is the one where OCT is less useful during all the operative phases. This is why the assessment of the patient eye must be performed by using other diagnostic instruments that allow the evaluation of posterior chamber anatomy. OCT alone is not sufficient to properly examine this site.

Because there is no specific literature regarding the use of anterior segment investigation techniques in this type of surgery, the data of our clinic have been thoroughly analysed. During the preoperative check-up, all our patients perform specific instrumental tests; all those patients suffering from IOL subluxation or those who require SFIOL positioning perform both an AS-OCT and an OCULUS PENTACAM exam.

It is of vital importance, during the preoperative examinations performed a week before surgery, to have registered keratometric measurements in two different days [39]. Objective measurement methods such as Pentacam topography and keratometry of the IOL Master should be performed and always compared with each other to reduce the possibility of any mistake and to collect accurate and repeatable data. During the aforementioned preoperative examinations, OCT images of the anterior segment have to be analysed, and 3D reconstruction could be performed by using OCULUS PENTACAM. In the images, it can easily be noticed the dislocation of the lens in both the eyes, the measure of the pupil diameter which is of fundamental. The pronounced irregularity of the pupil, which might be seen in Fig. 1 for example, could be due to the patient’s chronic therapy with dutasteride and silodosin [40].

In the intraoperative and postoperative phase, OCT might be useful to analyse the position of the lens and its distance from a hypothetically classic IOL in the posterior chamber. Furthermore, it allows the evaluation of the haptics in the intrascleral site and, should the latter have been positioned in a pocket, it allows its stability during postoperative examinations [41]. There are three surgical moments that may require the use of AS-OCT integrated in the operating microscope [22]: the first is at the end of the preparation of the intrascleral pockets to check its deepening. At this moment, it is of vital important to verify that, on one hand, there is sufficient space to accommodate the SFIOL plugs, and, on the other, that this slot is not excessively large so as to cause rotation of the lens and its subsequent risk of release in the posterior chamber. The second intraoperative moment is at the end of the insertion of the lens: AS-OCT might be used to verify the positioning of the central plate compared with the iris in mydriasis and to verify the distance between the SFIOL and the cornea [22]. This distance affects the effective dioptric power of the lens and should correspond to the preoperative clinical data. Free attachment of a scleral fixation lens increases the risk of anisometropia [42]; therefore, a double-check should be performed. The third operative moment should aim to verify the correct positioning of the SFIOL haptics in the scleral pockets, at the end of the surgery, after the conjunctival suture.

Hence, scleral fixation lenses surgically involve both the anterior and posterior segments. The surgery involves an anterior vitrectomy, a possible cause of vitreoretinal complications such as the aggravation of a previous interface syndrome, macular pucker or the development of cystoid macular oedema [42]. Therefore, the pre-postoperative evaluation must always also investigate the posterior segment. OCT is certainly the best tool for this purpose. Furthermore, even from the organizational point of view of the structure that welcomes the patient, a single instrument allows a complete diagnostic analysis thanks to the exchange of a lens in the OCT. Further studies should be aimed at investigating the use of anterior and posterior segment OCT as a routine pre and postoperative diagnostic tool [42].

The ocular structure that can most easily be examined by using OCT of the anterior segment is certainly the cornea. In particular, it is not important to check not only the entry site used for the intervention but also the postoperative result with possible damage to all corneal layers. The OCT of the anterior segment in the pre-/intra- and postoperative phase has already proven its centrality for the interventions of PKP, DALK, DSAEK and DMEK [43]. In these interventions, the use of the intraoperative guide of the microscope and the OCT of the anterior segment can be fundamental to immediately evaluate corneal astigmatism using the various guide systems, red dots or optical viewfinders, for the correction of the sutures and the juxtaposition of the graft. These are all possible patient’s comorbidity that must be carefully evaluated and monitored in case of SFIOL surgery. In fact, the intervention not only involves the anterior and posterior chamber but also the stress to which the cornea is subjected to mediate the insertion of the lens that is greater compared with other phacoemulsification interventions, with the possible risk of corneal decompensation [44], the formation of leukomas or strong astigmatism. This is due both to the more complex technique and to the need for greater manual skills for the surgeon. Further studies will be needed that quantify the value of these tests as an intra- and postoperative aid to monitor the integrity of the corneal structures. In the postoperative phase, the evaluation by endothelial count and microscopy can add with quantitative precision the extent of the damages discussed.

**Fig. 3:**
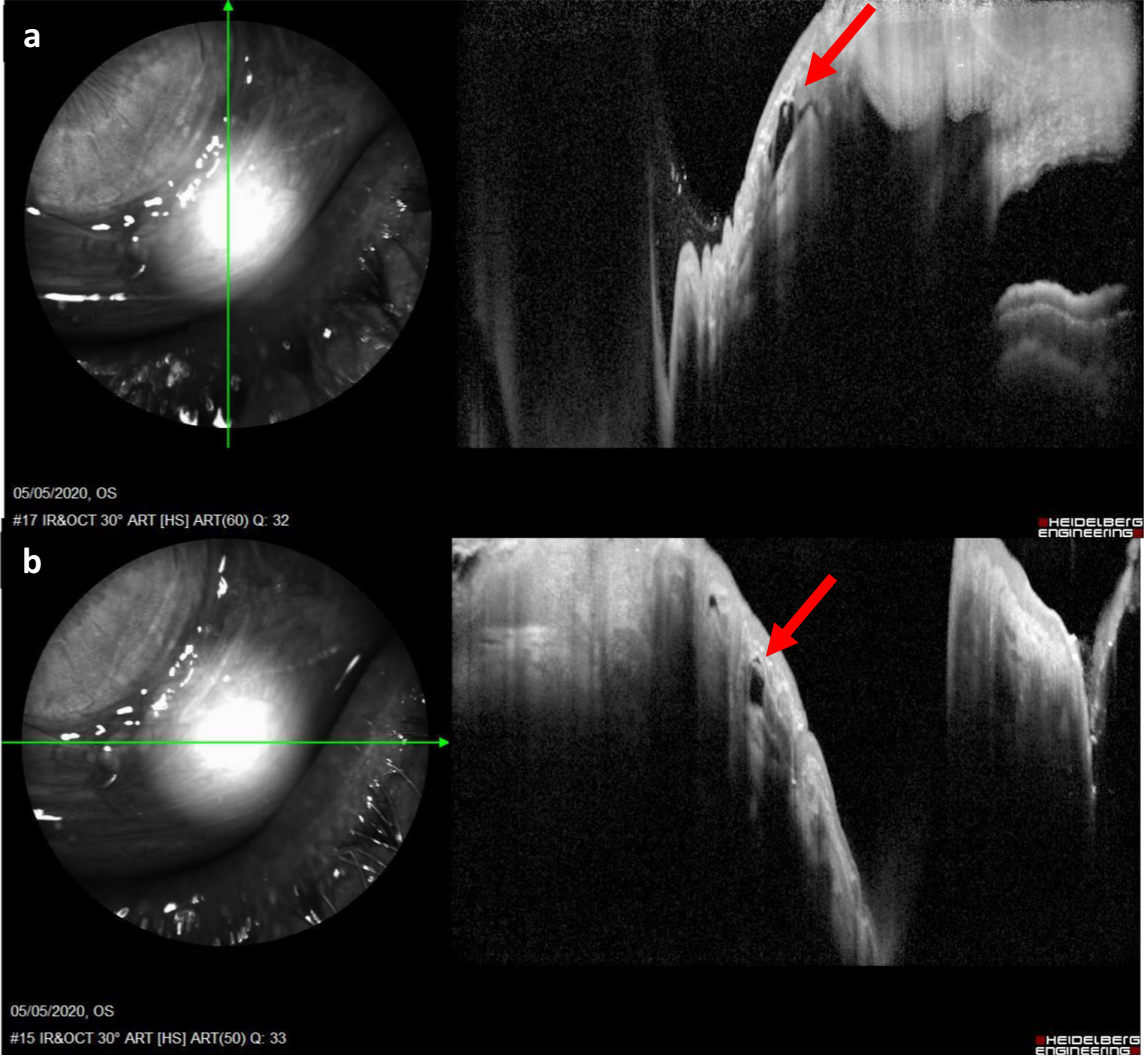
**a, b** Positioning of SFIOL plug-in in the intrascleral pocket. Red arrow indicates the haptic plug-in

When analysing the anterior segment complications that may result from cataract surgery, such as malposition of the lens, rupture of the plugs and angle closure, AS-OCT might be useful to carry out an objective evaluation, as it happens during the implantation of all the other IOLs [45]. The execution of postoperative tests supports the first and the following examinations, by searching for specific complications of this intervention. It is important to verify using AS-OCT, the correct deepening of the haptics in the intrascleral pockets, their perpendicular position to scleral incision, the definitive orientation of the lens and its distance from the iris plane [46] (Fig. 3a, b).

**Fig. 4:**
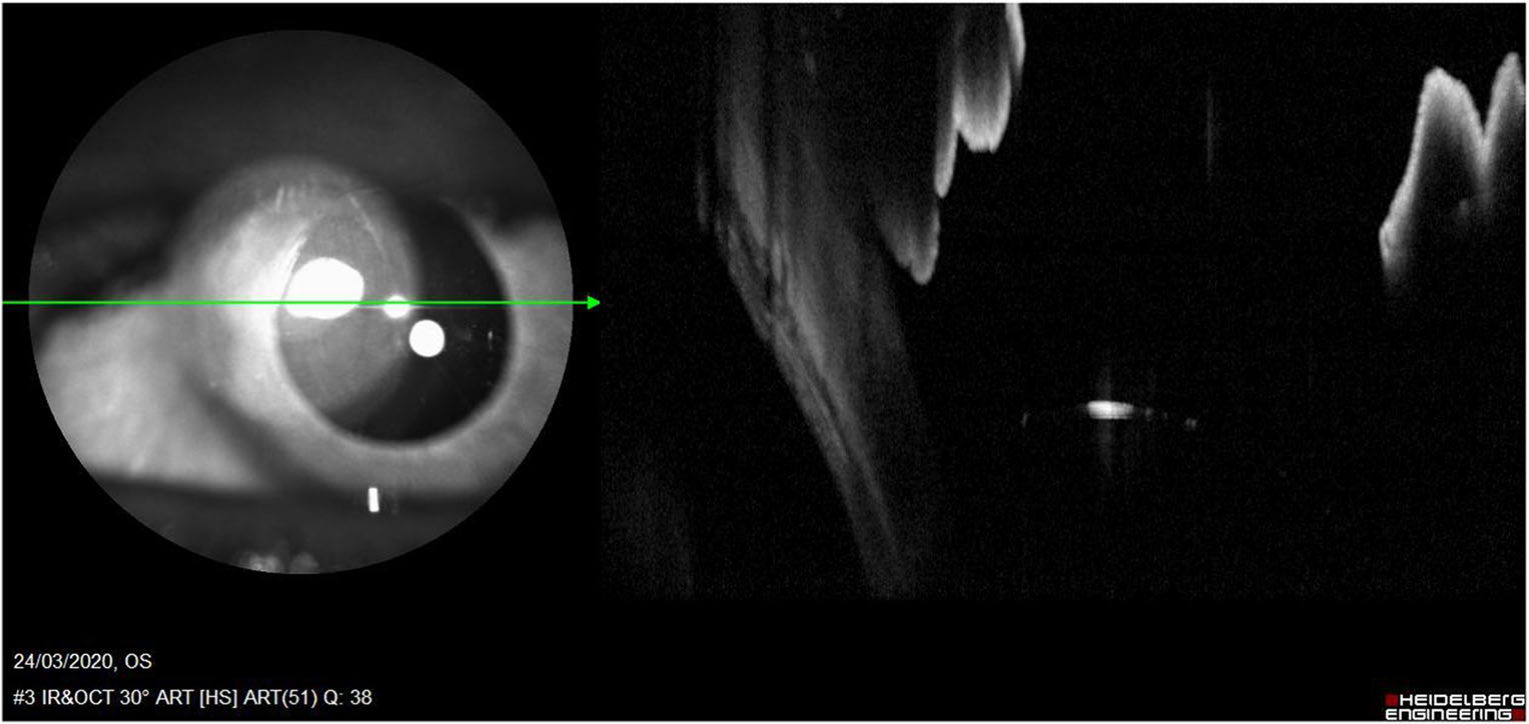
Correct SFIOL implantation: SFIOL is slightly visible, unambiguous evaluation cannot be completed with AS-OCT

**Fig. 5:**
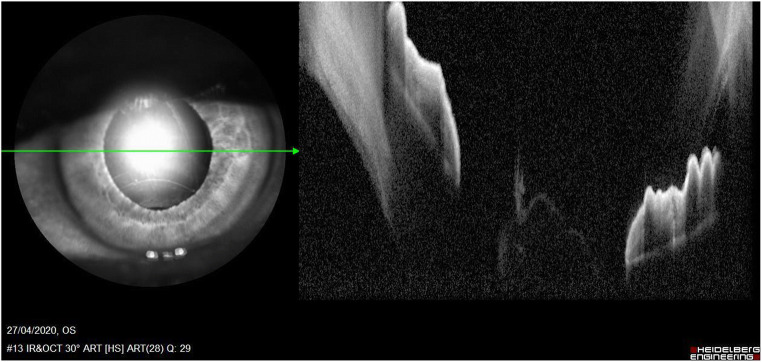
Incorrect SFIOL implantation after plug-in haptic rupture. Posterior chamber is not evaluable

During postoperative examinations, the limits of these techniques are more disabling. In fact, even after surgery is performed, all the impediments that do not allow a complete examination of the posterior chamber are maintained. Postoperative images are not clear, with current technologies, and do not help sufficiently to identify complications such as displacement, non-centring or stability of the IOL (Figs. 4 and 5).

IOL’s dislocation and eventually the rupture of capsular bag have been increasing due to the always swift surgery and to the aggressiveness of the ultrasounds used. Currently, in literature, there is not a preventive diagnostic evaluation technique that might be used as postoperative phacoemulsification follow-up in patients who have undergone cataract surgery. Hence, the need to perform surgery only when the disease is manifested. The improved survival of the patients and the surgical characteristics that have been described are one of the major causes of the increase of implants of scleral fixation lenses. Nevertheless, such increase has been faster than the studies published in literature concerning the risks, the possible complications and the correct surgical technique of implants. Fixation of SFIOL 3-Piece through prolene sutures has a limited in vivo duration [47]; the insertion of the lens assumes a corneal incision of 7 mm on average, with possible consequent postoperative astigmatism. Glue has not ensured an efficient stability for fixation [48]. The use of suspension models with haptics plug-in might represent a marked improvement, with constant results over time [49]. Nevertheless, its positioning and follow-up must be supported by the use of an efficient intra- and postoperative AS-OCT evaluation.

## Conclusions

Technological development reaches its climax when the best current surgical technique is supported by new types of lenses and new pieces of machinery. The former makes the surgeon’s job easier and thus more accurate; the latter makes it possible for the surgeon to rely on diagnostic imaging at any time. Intraoperative OCT of the anterior segment ensures the objectification of the anatomic result at the end of the procedure. By ensuring the development of a flawless 3D reconstruction of the patient’s eye structures, by allowing a close intraoperative monitoring of the procedure and finally a detailed monitoring during follow-up examinations, modern technology may be the key to maximize the precision and the efficacy of all surgical procedures. Nevertheless, postoperative images are not, with current technologies, clear and do not help sufficiently to identify complications such as displacement, non-centring or stability of a sutureless intrascleral fixation IOL with plug-in fixation.

## Data Availability

Not applicable.

## References

[1] Huang D, Swanson EA, Lin CP, Schuman JS, Stinson WG, Chang W (1991). Optical coherence tomography. Science (80- ).

[2] Ramos JLB, Li Y, Huang D (2009). Clinical and research applications of anterior segment optical coherence tomography - a review. Clin Exp Ophthalmol.

[3] Lansingh VC, Carter MJ, Martens M (2007). Global cost-effectiveness of cataract surgery. Ophthalmology..

[4] Nguyen P, Huang D, Li Y, Sadda SR, Ramos S, Pappuru RR (2012). Correlation between optical coherence tomography-derived assessments of lower tear meniscus parameters and clinical features of dry eye disease. Cornea.

[5] Tang M, Wang L, Koch DD, Li Y, Huang D (2012). Intraocular lens power calculation after previous myopic laser vision correction based on corneal power measured by Fourier-domain optical coherence tomography. J Cataract Refract Surg.

[6] Piñero DP, Plaza Puche AB, Alió JL (2008). Corneal diameter measurements by corneal topography and angle-to-angle measurements by optical coherence tomography: evaluation of equivalence. J Cataract Refract Surg. Jan.

[7] Piñero DP, Plaza AB, Alió JL (2008). Anterior segment biometry with 2 imaging technologies: very-high-frequency ultrasound scanning versus optical coherence tomography. J Cataract Refract Surg.

[8] Creese K, Ong D, Zamir E (2012) Should macular optical coherence tomography be part of routine preoperative cataract assessment? Clin Exp Ophthalmol 40(1):e118–9. 10.1111/j.1442-9071.2011.02623.x 21668777

[9] Schallhorn JM, Tang M, Li Y, Song JC, Huang D (2008). Optical coherence tomography of clear corneal incisions for cataract surgery. J Cataract Refract Surg.

[10] Wang L, Dixit L, Weikert MP, Jenkins RB, Koch DD (2012). Healing changes in clear corneal cataract incisions evaluated using Fourier-domain optical coherence tomography. J Cataract Refract Surg.

[11] Palanker DV, Blumenkranz MS, Andersen D, Wiltberger M, Marcellino G, Gooding P et al (2010) Femtosecond laser-assisted cataract surgery with integrated optical coherence tomography. Sci Transl Med 2(58):58ra85. 10.1126/scitranslmed.3001305 21084720

[12] Nagy ZZ, Ecsedy M, Kovács I, Takács Á, Tátrai E, Somfai GM (2012). Macular morphology assessed by optical coherence tomography image segmentation after femtosecond laser-assisted and standard cataract surgery. J Cataract Refract Surg.

[13] Alió JL, Piñero DP, Sala E, Amparo F (2010). Intraocular stability of an angle-supported phakic intraocular lens with changes in pupil diameter. J Cataract Refract Surg.

[14] Buehl W, Findl O (2008). Effect of intraocular lens design on posterior capsule opacification. J Cataract Refract Surg.

[15] Carreño E, Portero A, Galarreta DJ, Merayo JM (2012). Interface fluid syndrome associated with cataract surgery. J Refract Surg.

[16] Hodge C, Bali SJ, Lawless M, Chan C, Roberts T, Ng D (2012). Femtosecond cataract surgery: a review of current literature and the experience from an initial installation. Saudi J Ophthalmol.

[17] Chang D (2006) Ophthalmology ED-CO in. Phakic intraocular lenses. Indian J Ophthalmol 57(2):165–169.

[18] Kumar DA, Agarwal A, Prakash G, Jacob S, Saravanan Y, Agarwal A (2011) Evaluation of intraocular lens tilt with anterior segment optical coherence tomography. Am J Ophthalmol 151(3):406–12.e2. 10.1016/j.ajo.2010.09.013 21236406

[19] Pérez-Cambrodí RJ, Piñero DP, Blanes-Mompó FJ, Ferrer-Blasco T, Cerviño A (2012). Preliminary in vivo positional analysis of a posterior chamber phakic intraocular lens by optical coherence tomography and its correlation with clinical outcomes. J Optom.

[20] Werner L, Michelson J, Ollerton A, Leishman L, Bodnar Z (2012). Anterior segment optical coherence tomography in the assessment of postoperative intraocular lens optic changes. J Cataract Refract Surg.

[21] Nguyen P, Chopra V (2013). Applications of optical coherence tomography in cataract surgery. Curr Opin Ophthalmol.

[22] Lytvynchuk LM, Glittenberg CG, Falkner-Radler CI, Neumaier-Ammerer B, Smretschnig E, Hagen S et al (2016) Evaluation of intraocular lens position during phacoemulsification using intraoperative spectral-domain optical coherence tomography. J Cataract Refract Surg. 42(5):694–702. Elsevier Inc 10.1016/j.jcrs.2016.01.044 27255245

[23] Almutlak M, Aloniazan T, May W (2017). Real-time optical coherence tomography incorporated in the operating microscope during cataract surgery. Middle East Afr J Ophthalmol.

[24] Das S, Kummelil MK, Kharbanda V, Arora V, Nagappa S, Shetty R (2016). Microscope integrated intraoperative spectral domain optical coherence tomography for cataract surgery: uses and applications. Curr Eye Res.

[25] Titiyal JS, Kaur M, Shaikh F, Goel S, Bageshwar LMS (2020). Real-time intraoperative dynamics of white cataract-intraoperative optical coherence tomography-guided classification and management. J Cataract Refract Surg.

[26] Davis D, Brubaker J, Espandar L, Stringham J, Crandall A, Werner L (2009). Late in-the-bag spontaneous intraocular lens dislocation. Evaluation of 86 consecutive cases. Ophthalmology.

[27] Smiddy WE, Ibanez GV, Alfonso E, Flynn HW (1995). Surgical management of dislocated intraocular lenses. J Cataract Refract Surg.

[28] Fernández-Buenaga R, Alio JL, Pérez-Ardoy AL, Larrosa-Quesada A, Pinilla-Cortés L, Barraquer R (2013). Late in-the-bag intraocular lens dislocation requiring explantation: risk factors and outcomes. Eye.

[29] Al-Halafi AM, Al-Harthi E, Al-Amro S, El-Asrar AA (2011). Visual outcome and complications of pars plana vitrectomy for dislocated intraocular lenses. Saudi J Ophthalmol.

[30] Hayashi K, Hirata A, Hayashi H (2007). Possible predisposing factors for in-the-bag and out-of-the-bag intraocular lens dislocation and outcomes of intraocular lens exchange surgery. Ophthalmology.

[31] Hayashi K, Ogawa S, Ichi MS, Hirata A, Yoshimura K (2016). A classification system of intraocular lens dislocation sites under operating microscopy, and the surgical techniques and outcomes of exchange surgery. Graefes Arch Clin Exp Ophthalmol.

[32] Karadag R, Celik HU, Bayramlar H, Rapuano CJ (2016). Sutureless intrascleral fixated intraocular lens implantation. J Refract Surg.

[33] Can E (2018). Flapless and sutureless intrascleral fixation of posterior chamber intraocular lens for correction of aphakia. J Cataract Refract Surg.

[34] Veronese C, Maiolo C, Armstrong GW, Primavera L, Torrazza C, Della Mora L (2020). New surgical approach for sutureless scleral fixation. Eur J Ophthalmol.

[35] Kohnen T, Thomala MC, Cichocki M, Strenger A (2006). Internal anterior chamber diameter using optical coherence tomography compared with white-to-white distances using automated measurements. J Cataract Refract Surg.

[36] Shen P, Ding X, Congdon NG, Zheng Y, He M (2012). Comparison of anterior ocular biometry between optical low-coherence reflectometry and anterior segment optical coherence tomography in an adult Chinese population. J Cataract Refract Surg.

[37] Warrier S, Wu HM, Newland HS, Muecke J, Selva D, Aung T (2008). Ocular biometry and determinants of refractive error in rural Myanmar: the Meiktila eye study. Br J Ophthalmol.

[38] Yoo YS, Whang WJ, Kim HS, Joo CK, Yoon G (2019) Preoperative biometric measurements with anterior segment optical coherence tomography and prediction of postoperative intraocular lens position. Med (United States) 98(50): e18026. 10.1097/MD.0000000000018026 PMC6922509 31852065

[39] Turhan SA, Yigit DD, Toker E (2019) Corneal epithelial thickness and corneal curvature changes during the day: the effects of daily disposable contact lens wear. Contact Lens Anterior Eye. 43(4):389–394. 10.1016/j.clae.2019.11.017 31836203

[40] Ozcura F, Irgat SG (2020). Bilateral intraoperative floppy iris syndrome associated with silodosin intake. Eurasian J Med.

[41] Kucumen RB, Yenerel NM, Gorgun E, Kulacoglu DN, Dinc UA, Alimgil ML (2008). Anterior segment optical coherence tomography measurement of anterior chamber depth and angle changes after phacoemulsification and intraocular lens implantation. J Cataract Refract Surg.

[42] Davies EC, Pineda R (2018). Complications of scleral-fixated intraocular lenses. Semin Ophthalmol.

[43] Ziaei M, Vellara HR, Gokul A, Ali NQ, McGhee CNJ, Patel DV (2020). Comparison of corneal biomechanical properties following penetrating keratoplasty and deep anterior lamellar keratoplasty for keratoconus. Clin Exp Ophthalmol.

[44] Williams DL (1996). Corneal decompensation after cataract surgery. Ophthalmology.

[45] Wang X, Dong J, Wang X, Wu Q (2013) IOL tilt and decentration estimation from 3 dimensional reconstruction of OCT image. PLoS One 8(3):e59109. 10.1371/journal.pone.0059109 PMC3598664 23554982

[46] Batur M, Seven E, Tekin S, Yasar T (2017). Anterior lens capsule and iris thicknesses in pseudoexfoliation syndrome. Curr Eye Res.

[47] Wasiluk E, Krasnicki P, Dmuchowska DA, Proniewska-Skrętek E, Mariak Z (2019). The implantation of the scleral-fixated posterior chamber intraocular lens with 9/0 polypropylene sutures – long-term visual outcomes and complications. Adv Med Sci.

[48] Ragam A, Ritterband DC, Waisbren EC, Mathew-Padiyedathu J, Kang J, Seedor JA (2018). Clinical outcomes and intraocular pressure control after scleral-glued intraocular lens insertion in eyes with pseudoexfoliation. J Glaucoma.

[49] Rossi T, Iannetta D, Romano V, Carlevale C, Forlini M, Telani S et al (2020) A novel intraocular lens designed for sutureless scleral fixation: surgical series. Graefes Arch Clin Exp Ophthalmol. 259(1):257–262. 10.1007/s00417-020-04789-3 32529278

